# Diagnosis and Treatment of Lumbar Giant Cell Tumor of the Spine: Update on Current Management Strategies

**DOI:** 10.3390/diagnostics12040857

**Published:** 2022-03-30

**Authors:** Andrew R. Leggett, Ari R. Berg, Heidi Hullinger, Joseph B. Benevenia

**Affiliations:** 1 Department of Orthopaedics, Rutgers New Jersey Medical School, Newark, NJ 07103, USA; arb221@njms.rutgers.edu (A.R.B.); benevejo@njms.rutgers.edu (J.B.B.); 2 New Jersey Spine Specialists, Summit, NJ 07901, USA; hhullinger@njss.net

**Keywords:** giant cell tumor, lumbar spine, diagnosis, treatment, denosumab, en bloc spondylectomy

## Abstract

(1) Background: Giant Cell Tumor of the spine remains a difficult tumor to treat. Recent advances in adjuvant therapy such as denosumab and innovations in surgical technique in the last 5 years have given providers new options for treatment after a successful diagnosis of the tumor. (2) Methods: Articles published between 1927 and 2021 were selected from PubMed and Scopus searches using key words “Giant Cell Tumor” AND “Lumbar Spine” AND “Treatment”. Relevant articles were reviewed and selected by the authors. (3) Results: A total of 191 articles were discovered. Complete en bloc spondylectomy remains the most definitive treatment option; however, this surgery is challenging and carries a high rate of complication. New adjuvant therapies including denosumab offer a viable alternative to surgery. (4) En bloc spondylectomy remains the gold standard treatment for Giant Cell Tumor of the spine with the lowest published recurrence rate. The use of (neo)adjuvant denosumab improves recurrence rates. More data are needed to determine if denosumab alone is a viable standalone definitive treatment.

## 1. Introduction

Giant Cell Tumors of the lumbar spine (GCTS) are a locally aggressive benign tumor with the potential to undergo malignant transformation [1]. Early presentation often includes nonspecific complaints of lower back pain and lower extremity neurologic symptoms [2]. The incidence of first-time low back pain episodes in the general population ranges from 6.3% to 15.4% with incidence peaking in the 3rd decade of life [3]. GCTS represents a rare, but easily diagnosable, cause of low back pain which can be treated with conservative or invasive measures. The diagnosis of these tumors can often be suspected based on characteristic imaging features and ultimately confirmed with a biopsy [4]. Historically, these tumors are treated with en bloc spondylectomy vs. intralesional resection of the affected vertebral elements [5,6,7,8,9,10]. Recent advances in surgical technique and the use of denosumab, a monoclonal antibody that binds the cytokine RANKL, are facilitating the removal of tumors previously considered inoperable [11,12,13,14,15]. Furthermore, denosumab alone is being explored as a potential non-operative treatment for GCTS, particularly for those whose resections would carry high morbidity [11,15,16].

The goal of this paper is to review recent and classical literature regarding GCTS in order to development an algorithm for the diagnosis of GCTS in the mobile lumbar spine and to provide an update on treatment strategies developed within the last 5 years.

## 2. Materials and Methods

Two independent reviewers (AL, AB) performed a search of all peer-reviewed relevant literature in English published between 1927 and 2021. The electronic databases queried include Scopus and PubMed using keywords “Giant Cell Tumor” AND “Lumbar Spine” AND “Treatment”. Additional relevant articles were obtained from the reference lists of the articles retrieved from the initial database search.

Criteria for inclusion included articles which covered the biology, diagnosis, and management of Giant Cell Tumors of the lumbar spine. Articles were excluded if they did not reference GCTS, or exclusively focused on GCTS in the sacrum, thoracic, or cervical spine. Two authors (AL, AB) independently reviewed abstracts after all duplicate articles were removed. All selected articles underwent a full paper review of all papers which met the inclusion criteria, and final selection was determined through discussion for consensus.

## 3. Results

A total of 191 peer reviewed publications were selected for review through a database query (142 PubMed, 112 SCOPUS), and 63 articles were found in both database queries. Therefore, a total of 191 articles were selected for full paper review. Of these articles, 49 were selected for inclusion. An additional 12 articles were discovered in the reference list of the initial 49 articles selected. Nine of these articles were included in the final bibliography. Levels of evidence of the selected articles are included in Appendix A.

## 4. Discussion

### 4.1. Epidemiology

The Giant Cell Tumor (GCT) is a well described primary bone tumor which frequently occurs in the appendicular skeleton in the epiphysis and metaphysis of the long bones with a predominance for the distal femur, proximal tibia, and distal radius [16]. A recent nationwide pathology registry study from the Netherlands placed the incidence of GCT of the bone at 1.7 per million inhabitants per year with a male to female ratio of 1:1.38 [17]. Overall incidence of Spinal Giant Cell Tumor (SGCT) is cited between 2 and 15% of all GCT with a predominance for the sacral region, and most commonly presents in the 3rd and 4th decades of life [5,6,18,19,20,21]. GCT is known as a relatively rare primary tumor of the bone, with an even lower incidence of cases presenting in the mobile spine defined as above the sacrum at a rate of 6.5% of all primary GCT according to the Mayo Clinic [20]. The incidence of GCTS can be further broken down by location within the mobile spine. GCTS occurred in the lumbar spine in 16–52% of cases reported in the six largest case series referenced within this paper [5,6,7,8,10,22]. No large database studies exist to quantify the prevalence/incidence of GCTS by location within the spine at this time.

GCT is commonly described as a benign tumor with locally aggressive behavior. Recurrence after treatment is estimated to be as high as 50%. GCT converts to a malignant pathology in 1–3% of cases and metastasizes to the lungs in as high as 5% of cases [1,23]. However, literature regarding SGCT indicates that there is a higher rate of metastasis to the lungs. A review of 51 cases of SGCT performed by Donthineni et al. showed 14% of SGCT metastasized to the lungs, significantly higher than the 5% previously reported in GCT literature [24]. The locally destructive nature and risk of malignant transformation necessitates aggressive treatment of GCT. This necessity is compounded in the mobile spine, where compression places neural elements at risk, leading to more severe complications including even Cauda Equina Syndrome.

### 4.2. Diagnosis

#### 4.2.1. Clinical Presentation

Patients with GCT of the mobile spine often present with pain and neurologic deficit of the lower extremity. As previously described, the typical patient is likely to be female in the 3rd or 4th decade of life. While rare, pediatric Giant Cell Tumor of the spine has been described in literature, and so this differential diagnosis should not be ruled out in younger patients [22]. The local aggressive nature of GCT leads to cortical destruction and expansion into soft tissues surrounding the tumor. In the spine, this puts neural elements at significant risk for compromise. Si et al. showed 8/10 patients with SGCT of the lumbar spine experienced radicular symptoms ranging from radiating pain down the extremity to pain with lower extremity weakness [25]. Severe cases of neurologic compression due to SGCT have been reported by Randhawa et al. to lead to Cauda Equina Syndrome. Interestingly, full neurologic recovery was obtained in both cases with denosumab treatment alone [26].

Lower back pain and neurologic compromise are the presenting symptoms for a wide range of spinal pathology including tumor and non-tumor conditions. Therefore, the first step in the diagnosis of SGCT should include a thorough history and physical followed by a radiographic evaluation of the lumbar spine.

#### 4.2.2. Imaging

Imaging studies are critical for the development of a differential diagnosis in the setting of low back pain and neurologic deficits. The combination of plain radiograph, CT scan, and MRI, in a patient in the typical age range, can lead to a presumptive diagnosis of GCT of the lumbar spine prior to definitive biopsy. Shi et al. performed a 34-case review of radiographic presentation of GCT which noted that 85% of tumors originated in the vertebral body and extended into the posterior elements of the spine, while 15% of tumors originated in the posterior elements and extended into the vertebral body [27]. This is an important distinction as the majority of spinal column tumors such as aneurysmal bone cyst (ABC), osteoid osteoma, and osteoblastoma frequently affect only the posterior elements of the spine [16]. Si et al. discovered a similar pattern in GCT of the spine arising from the vertebral body with expansion of the tumor into pedicle and posterior elements in 13/18 cases [25]. The lesions can present with central or eccentric origin in the vertebral body [25,27,28]. The aggressive nature of the benign tumor leads to local expansion both into the spinal canal (57–92% of cases [25,27]) and paravertebral tissue (68% of cases [27]).

#### 4.2.3. Plain Radiograph

**Figure 1:**
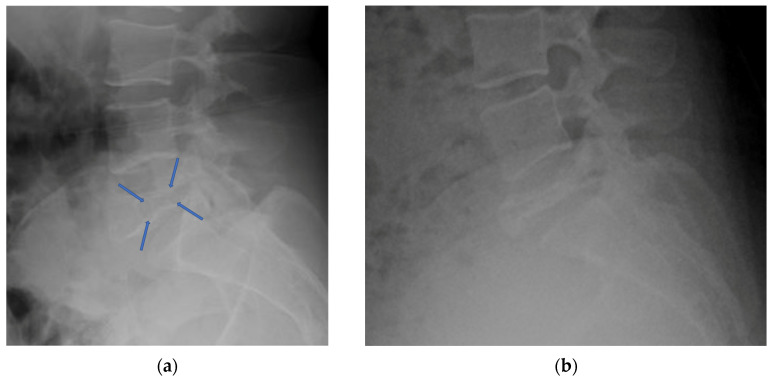
(**a**) Lateral radiograph of a biopsy confirmed L5 Giant Cell Tumor; arrows indicate characteristic soap bubble appearance in the L5 vertebral body with loss of vertebral body height indicating pathologic vertebral body fracture. (**b**) Lateral radiograph of the same L5 Giant Cell Tumor after 6 months of IV denosumab treatment showing consolidation of the tumor.

The first form of imaging that should be obtained is the plain radiograph. The results of these studies will determine the need and direction of future advanced imaging. GCTS displays several distinct radiographic characteristics and will guide future imaging and treatment decisions. GCT of the spine can present either in a typical “soap bubble” appearance on plain radiograph or as a purely lytic lesion [25,27]. The aggressive and lytic nature of the tumor can lead to varying degrees of vertebral compression ranging from mild to outright vertebral plana (Figure 1). Aggressive expansion into the surrounding soft tissue can be characterized on a plain radiograph as a soft tissue shadow usually present in the psoas muscle [27]. It is also possible to diagnose a pathologic fracture on plain film which was present in 5/5 lumbar GCT plain radiographs reviewed by Si et al. [25]. Plain radiographic findings, while useful, are insufficient to determine whether or not a biopsy of the lesion is indicated. The abnormal findings described above should push the diagnosing physician to obtain both a CT scan (with or without contrast) and full sequence MRI of the lumbar spine.

#### 4.2.4. Computerized Tomography Scan

**Figure 2:**
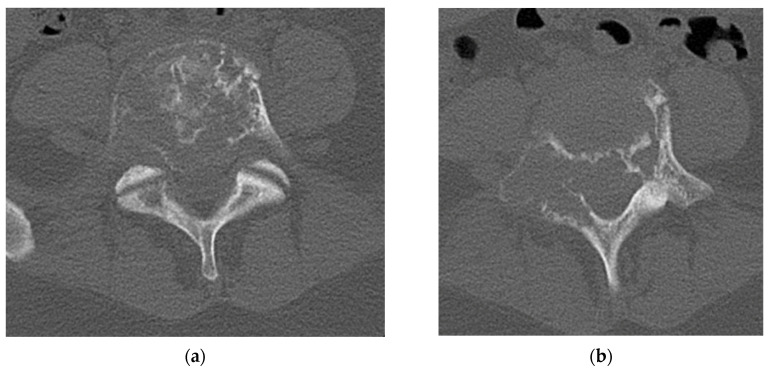

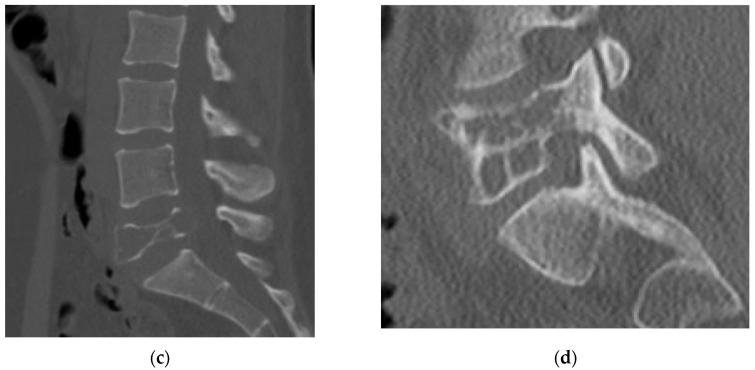
(**a**) Axial CT cuts of L5 vertebral body shows pseudotrabeculation of vertebral body with eccentric thinning of the right vertebral body cortex and formation of a contralateral sclerotic margin. (**b**) Bilateral pedicle involvement with near complete destruction of right sided L5 pedicle. (**c**) Sagittal cuts of L5 vertebral body shows loss of vertebral height indicating compression fracture with no retropulsion of bony elements. The lesion is seen to by lytic with cortical erosion especially in the posterior vertebral body. (**d**) Sagittal cut of L5 vertebral body demonstrates classic “soap bubble” imaging characteristic seen on the vertebral body of L5 on CT scan.

A CT scan of the lumbar spine provides a more detailed analysis of the bony remodeling which occurs during the formation of GCT. The “soap bubble” appearance seen on the plain radiograph is secondary to pseudotrabeculation, which can be appreciated on axial cuts of the CT scan [25]. The tumor itself closely resembles soft tissue density with no evidence of bony mineralization [27]. The cortex displays thinning or complete eradication, in which case there is an extension of the tumor into the surrounding soft tissue (Figure 2). A sclerotic border within the vertebral body is not uncommon (33%) and will be positioned opposite the eccentric distribution of the tumor. Shi et al. proposed that this unique appearance can be instrumental in the imaging diagnosis of a GCT [27].

#### 4.2.5. MRI

**Figure 3:**
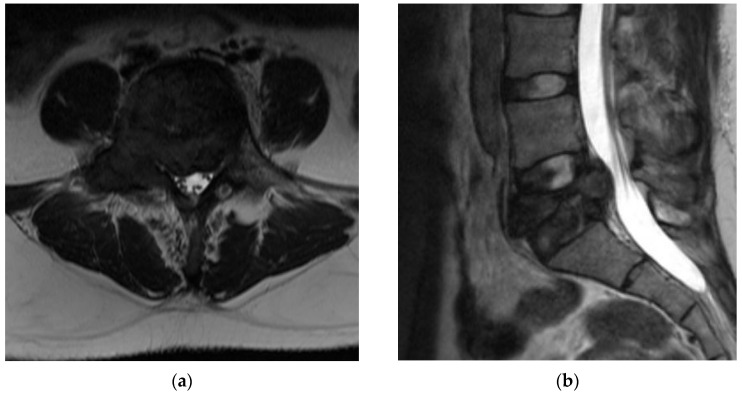
(**a**) Axial T2 weighted MRI cuts of L5 vertebral body show low signal intensity of the tumor body with extension into bilateral pedicles. There is mild compression of the spinal canal. (**b**) Sagittal T2 weighted MRI cuts of L5 vertebral body again show low signal intensity. There is retropulsion of the compressed L5 vertebral body causing narrowing of the canal.

The third recommended imaging modality is magnetic resonance imaging (MRI). MRI is useful to evaluate the neural elements as well as further evaluate the bony involvement. GCT presents with a heterogenous signal intensity on T2 weighted images. The solid components have a low signal intensity due to collagen and haemosiderin deposition [29]. As described by Kwon et al. this characteristic is not unique to GCT of the spine; however, it does help to distinguish GCT from other spinal tumors such as metastases, chordoma, and lymphoma, which typically present with high signal intensity on T2 weighted imaging [2]. Aoki et al. showed, in a review of 10 MRI of GCT of the spine, that haemosiderin deposition shows nodular, zonal, whorled, or diffuse low signal intensity on all sequence images with low intensity further exaggerated on T2 images (Figure 3). This pattern of haemosiderin deposition further supports the diagnosis of GCT on MRI [29]. The high intensity signal seen on T2 images may correspond to intralesional hemorrhage or the formation of a secondary aneurysmal bone cyst (ABC) [28]. Si et al. noted the presence of fluid-fluid levels in 23.8% of cases on T1 and T2 images indicating the formation of a secondary ABC [25]. Fluid-fluid levels were also seen on 34% of T1 and 24% of T2 sequences by Shi et al.; however, they attribute these findings as nonspecific [27]. GCT shows an enhancement on contrast enhanced MR and CT images due to the hypervascular nature of the tumor [27]. In short, there is no defining characteristic of MRI which indicates the diagnosis of GCT; however, this imaging modality is still useful for defining the extent of soft tissue expansion and the relationship of the tumor with intraspinal neural elements for surgical planning [2].

### 4.3. Final Diagnosis

While clinical presentation and imaging studies including plain radiograph, CT, and MRI can help narrow the differential diagnosis to include Giant Cell Tumor of the spine, a final diagnosis cannot be confirmed without tissue biopsy and histologic evaluation. After imaging, other benign aggressive tumors such as ABC of the spine cannot be completely ruled out. Biopsy can be performed as an open or CT guided procedure. However, a CT guided biopsy will require close coordination with the interventional radiologist. A CT guided biopsy represents a safe and accurate method to confirm a suspected diagnosis of GCTS [30]. Standard biopsy principles should be applied regardless of the choice of method. Plan the biopsy to ensure that the biopsy tract can be resected if histopathology indicates GCTS. There are instances in which an excisional biopsy may be indicated, such as when a tumor is small and entirely located within the vertebral body in the mobile spine, and thus best approached open via a retroperitoneal approach. In the lumbar spine, a transpedicular approach should be used. If the pedicle has evidence of disease on imaging studies, enter through the diseased pedicle. Cannulate the pedicle with a trocar and use a Jamshidi needle to obtain a core of bone. Any biopsy performed during urgent decompression of the spine should be sent for frozen section and results interpreted prior to proceeding with decompression. If a malignant tumor is diagnosed during the frozen section, take care to avoid contamination of the surrounding tissue.

**Figure 4:**
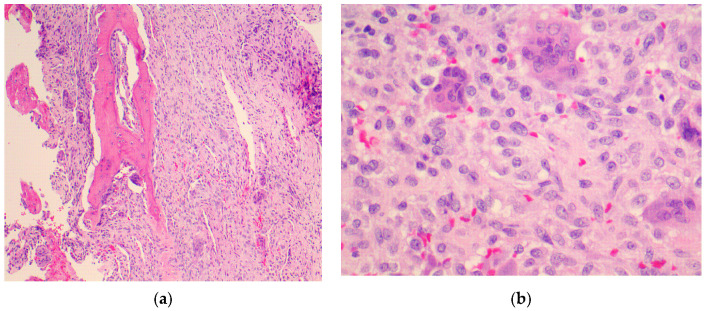
(**a**) Low magnification histologic examination of GCT of L5 vertebral body shows residual bone trabeculae with focal osteoid production. (**b**) High magnification histologic examination of a GCT of L5 vertebral body shows mononuclear cells interspersed with multinucleated giant cells.

Histologically, GCT of the spine presents as a spindle cell stroma with multiple multi-nucleated osteoclastic giant cells (Figure 4). Areas of recent hemorrhage with hemosiderin are also seen along with fibrous tissue that is high in collagen content [16]. A histologic examination is important to determine whether the tumor is benign or malignant. While rare, the malignant Giant Cell Tumor of the spine is a potentially devastating diagnosis which warrants aggressive treatment as outlined below. Histologically, Primary Malignant GCT of the bone (PMGCT) consists of normal Giant Cell Tumor areas interspersed by malignant pleomorphic spindle cells. Secondary Malignant Giant Cell Tumor of the bone (SMGCT) is a high grade sarcoma which arises at the previous site of treatment of a benign GCT after either radiotherapy, surgical excision, or both [31].

### 4.4. Classification and Staging

After diagnosis is confirmed with histology, staging of the tumor should be performed to aid in decisions regarding management of the tumor. Classically, the Enneking classification was used in SGCT classification. Boriani et al. developed a staging system specific to GCTS in 1997 which is still used today. They recognized that the original Enneking classification system, designed for musculoskeletal tumors of the long bones, had limitations when applied to the spine, which resulted in a disorganized approach to the management of tumors of the spine.

The Weinstein-Boriani-Biagnini Surgical Staging System sought to create a comprehensive surgical staging system for the management of spinal tumors; it classifies tumors by location in 12 radiating zones and five concentric layers. The longitudinal involvement of the tumor is described in the listed vertebral levels.

By classifying tumor involvement of the vertebral elements in this manner, surgeons can differentiate between three treatment choices: (1) vertebrectomy; (2) sagittal resection; and (3) resection of posterior arch. A vertebrectomy is appropriate if safe margins can be obtained while leaving one or both pedicles intact. Therefore, tumors confined to zones 4–8 or 5–9 can be treated in this manner. Sagittal resection is indicated when safe margins can be established for eccentric tumors of the vertebral elements which occupy zones 2–5 or 8–11. Resection of the posterior arch is indicated when safe tumor margins can be established for tumors occupying zones 3–10 [4].

GCTS are an aggressive benign tumor and at the time of staging often involve the majority, if not all, of the zones described by Boriani et al. Therefore, it is common that these tumors are removed with en bloc vs. total piecemeal spondylectomy to reduce recurrence.

#### 4.4.1. Lung Metastases and Malignant Transformation

The most common site of metastasis for GCT is the lungs with reported incidence ranging from 1 to 9% [32,33,34,35]. The rate of lung metastases in GCTS was shown to be higher at 13.7% in a retrospective case series of 51 patients published by Donthineni et al. [24]. Boriani had a similar incidence of lung metastases at 12% in a 49-patient case series [5]. Recurrence of the Giant Cell Tumor after treatment has been identified as a risk factor for pulmonary metastasis in studies relating to GCT of the long bone. Rock et al. found that recurrent GCT of the long bone was six times more likely to become metastatic to the lungs [36]. There is no current literature on the rate of lung metastasis in recurrent GCTS; however, suspicion should remain elevated for possible metastasis in recurrent cases due to an increased rate in recurrent GCT of the long bones.

A GCT of the bone is a benign tumor, but there is a risk for malignant transformation, especially after radiotherapy. The rate of conversion from benign to malignant GCT in the long bone following radiation has decreased with the advent of modern techniques such as image-guided intensity modulated radiotherapy and stereotactic radiosurgery [37]. Yin et al. published the largest cohort to date regarding the treatment of malignant Giant Cell Tumors of the spine with a total of 14 patients treated. Three cases were diagnosed as PMGCTS and the remaining 11 were SMGCTS. Three patients were treated with subtotal resection alone, three with total piecemeal resection, four with total piecemeal resection plus radiotherapy, and four were treated with total en bloc spondylectomy. The outcome of the subtotal resection alone showed a 100% recurrence rate, distant metastases in 1/3 cases, and mortality in 2/3 cases. Piecemeal resection alone carried a recurrence rate of 66.7%, metastatic rate of 33.3%, and mortality in 66.7% of cases. The piecemeal resection group with post-operative radiation showed recurrence in 50% of cases, distant metastases in 50%, and mortality in 50% of cases. The total en bloc spondylectomy group performed best with no recurrence, no distant metastases, and no mortality [31]. This small retrospective cohort is insufficient to yield a formal recommendation on the treatment of MGCTS, particularly as en bloc spondylectomy is often not possible when an entire vertebral segment is involved. However, it does highlight the severity of this disease process and the importance of aggressive treatment after diagnosis.

#### 4.4.2. Management of Giant Cell Tumor of the Lumbar Spine

The mainstay for treatment of GCTS of the lumbar spine is surgical management. Two surgical approaches, intralesional curettage and complete en bloc spondylectomy, have been used, and the results of both published in both historic and recent literature. The advent of denosumab as both an adjuvant and long-term treatment for GCT has both facilitated greater ease of surgical intervention as well as provided a viable alternative for patients who are not ideal surgical candidates. The section below will outline the risks and benefits of each proposed treatment method to aid future surgeons in the management of such a complex condition.

### 4.5. Denosumab

Denosumab is a fully human monoclonal antibody which targets the RANK ligand and prevents binding with the RANK surface receptor to inhibit differentiation, activation, and survival of osteoclast-like cells. Giant Cell Tumors of the bone have been shown to express RANKL, which contributes to the aggressive local destruction of bone seen in these tumors [12].

Given the role of RANKL in bone destruction caused by GCTB, a Phase II RCT was performed by Thomas et al. in 2010 to study the effect of denosumab on unresectable GCTB. In total, 30/35 (86%) of the patients evaluated experienced a tumor response after therapy. Response included 35/35 patients with near-elimination of giant cells upon repeat biopsy, and radiographic stabilization at 6 months in 10/15 patients deemed evaluable. The treatment was well-tolerated without serious adverse events which paved the way for denosumab as a viable treatment for GCT [12].

**Figure 5:**
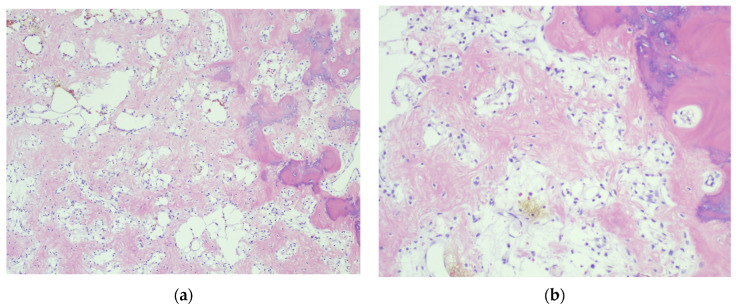
(**a**) Low magnification histologic examination of GCT of L5 vertebral body after 6 months of IV denosumab therapy shows increased ossification. (**b**) Mid-level magnification histologic examination of a GCT of L5 vertebral body shows decreased number of multinucleated giant cells with increased ossification and fibrosis.

Yamaya et al. published a case report to evaluate the histologic response of a GCT of the lumbar spine after 10 monthly cycles of denosumab. A micrograph evaluation of pre-denosumab biopsy samples showed numerous multinucleated giant cells interspersed in a background of neoplastic mononuclear stromal cells. Immunohistochemical staining revealed RANK-positive mononuclear cells and cyclooxygenase-2 (COX-2) positive mononuclear stromal cells. After treatment with denosumab and resection, a micrograph of tissues harvested showed a fibrous matrix with no multinucleated giant cells (Figure 5). Immunohistochemical staining showed the presence of RANK around woven bone, but no RANK or COX-2 positive cells [14].

Denosumab has been used in the treatment of GCTS as both an adjuvant (which will be explored in detail below) and as a stand-alone treatment. In 2015, Goldschlager et al. demonstrated that denosumab was an effective treatment in GCTS in a five-patient case series. All patients had radiographic responses to denosumab, and 4/5 had a confirmed histologic response. In total, 4/5 patients subsequently underwent en bloc or piecemeal resection of the tumor; the 5th patient underwent arthrodesis alone and her disease remained progression free at 24 months with no adverse events related to denosumab treatment [38].

**Figure 6:**
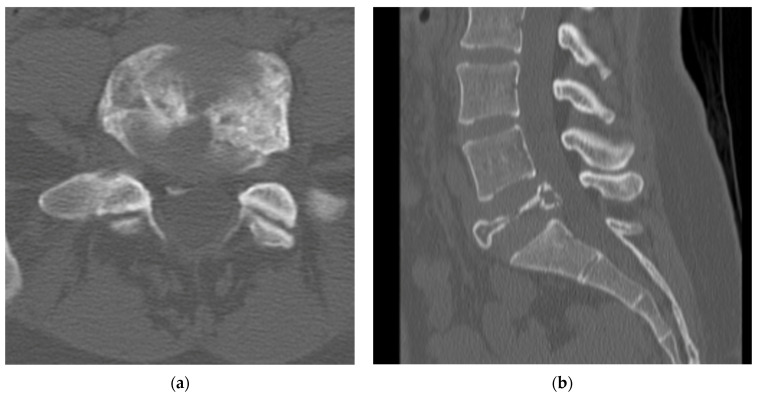
(**a**) L5 Giant Cell Tumor after 6 months of IV denosumab treatment. Axial cuts of L5 vertebral body show significant ossification of tumor with reestablishment of previously eroded cortices. (**b**) Sagittal cuts of L5 Giant Cell Tumor after 6 months of IV denosumab therapy show increased loss in vertebral height from previous CT scan; however, cortical margins are reestablished. There appears to be mild retropulsion of L5 vertebral body.

**Figure 7:**
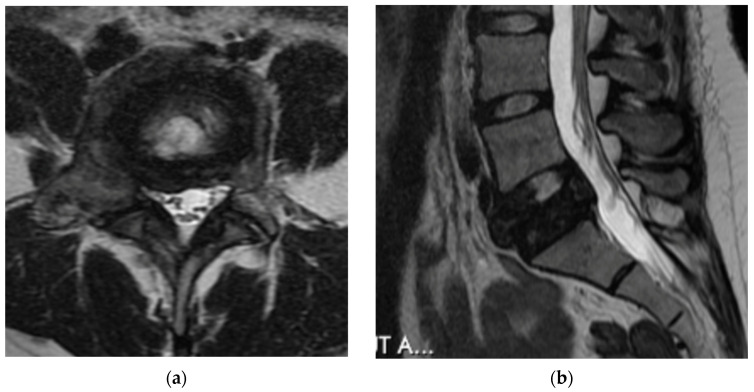
(**a**) L5 Giant Cell Tumor after 6 months of IV denosumab treatment. T2 weighted axial MRI cuts of L5 vertebral body show normalized bone marrow signal intensity when compared to pre-denosumab imaging. (**b**) Sagittal T2 weighted cuts of L5 Giant Cell Tumor after 6 months of IV denosumab therapy show reduction of retropulsion into the canal likely secondary to consolidation of tumor.

More recently, denosumab has been explored as a stand-alone treatment for GCTS with good results. Boriani et al. published a 10-patient case series which explored the role of denosumab as a stand-alone, pre-operative, and post-operative treatment. Three patients treated with denosumab alone had complete resolution of pain. Furthermore, 2/3 patients had a reduction in tumor size, with one patient experiencing no reduction in tumor size. Therapy duration included 83, 88, and 57 months with no adverse events related to denosumab treatment [11]. Mattei et al. published a case report detailing the treatment of a 22 yo female patient with a C2 GCT who underwent C1–C4 in situ stabilization and long-term denosumab therapy consisting of 26 months of follow up. After 26 months, there was no progression of disease and a radiographic evaluation showed a decrease in tumor size and formation of new cortical bone [39] (Figure 6 and Figure 7. In 2016, Randhawa et al. treated two patients with documented Cauda Equina Syndrome secondary to L5 GCTS with denosumab alone. Neither patient reported any adverse events secondary to denosumab treatment. One patient progressed from 4/5 to 5/5 muscle strength and completely regained bowel and bladder control after 2 weeks of treatment. The second patient progressed from 0/5 to 4/5 muscle strength after 3 months of treatment [26]. Yonezawa et al. studied the morphologic changes of four GCTS before and after denosumab therapy through an evaluation of CT scans. They found that osteolytic tumor volume decreased by 87% on average with a progressive loss of vertebral height in three of four cases. The one case without loss of vertebral height was attributed to a more robust anterior cortical rim. Interestingly, the average area of the spinal canal occupied by the tumor decreased from 56.1% to 15.1%, which is in accordance with the improvement in neurological symptoms after denosumab therapy seen in previously cited studies [15].

These studies highlight the potential of denosumab as a stand-alone treatment in GCTS; however, there is still no agreement on duration of the treatment. It is unclear if lifelong suppression would be required as a definitive treatment. Such duration of the treatment would raise the risk of adverse events such as osteonecrosis of the mandible [40].

#### 4.5.1. Radiation Therapy

While surgery remains the gold standard treatment, the complete resection of GCTS is technically challenging with the potential for spinal cord injury; therefore, a number of adjuvant therapies have been proposed to reduce the rate of recurrence. In addition to denosumab, adjuvants include radiation therapy (RT), selective arterial embolization, cryotherapy, ABC, bisphosphonates, and interferon alpha. Overall, data on the effectiveness of these adjuvant therapies are sparse. Here, we detail the evidence available for use of radiation therapy as an adjuvant for GCTS treatment.

Radiation therapy in the setting of GCTS can be considered either as an adjuvant or stand-alone treatment for patients with severe comorbidities or inoperable tumors [20]. While some studies have shown the effectiveness of RT in reducing recurrence, there are others that have shown conflicting evidence. Khan et al. reported the long-term results of the treatment of six patients diagnosed with GCTS who were treated with conservative surgery (consisting of either biopsy or subtotal resection) and radiotherapy. With a mean follow up of 13 years, five out of the six patients lived with no evidence of disease. One patient was alive with clinically asymptomatic disease [41]. Similarly, Sharma et al. published on six patients with cervical GCTS that they treated with subtotal resection and RT. At a mean follow up of 2 years, no patients showed a recurrence of disease [42]. Finally, Chakravarti et al. reviewed 20 patients with GCT, 12 of whom had GCTS, who were treated with a single course of megavoltage radiation (forty to seventy gray administered at 1.8 to 2.0 gray per fracture with an average total duration of five to seven weeks). With a median follow up of 9.3 years, the tumor had not progressed in 17 of the twenty patients (85%). Furthermore, no radiation-induced tumors were observed [43].

In contrast to the above cited articles, a number of studies have shown no improvement in recurrence rates with RT. Xu et al. performed a retrospective analysis of 102 patients with GCTS and found no improvement in recurrence rates at 2 and 5 years [7]. Similarly, Ruggieri et al. found no effect on recurrence in 31 patients with sacral GCTS who underwent intralesional resection plus adjuvant RT [44]. Leggon et al. performed a literature review of 239 sacral and pelvic GCTS and found that larger radiation doses also resulted in no significant reduction in recurrence rates. The authors further performed a subgroup analysis looking only at sacral GCTS. They found that treatment with intralesional resection and adjuvant RT resulted in no improvement in recurrence rates compared to either resection alone or RT alone [45].

Important factors to consider with regard to RT and GCTS are the local side effects and radiation-induced sarcoma. RT techniques have evolved extensively over the last several decades, which may improve effectiveness against GCTS. Intensity-modulated radiotherapy (IMRT) uses reconstructive imaging to deliver optimal doses of radiation safely, with low dose exposure to surrounding structures. Roeder et al. treated five patients with IMRT for GCT—four of whom had GCT of the sacrum—not amenable to complete resection. With a medial follow up of 4 years, the overall survival was 100% and local control rate 80% [46]. A newer technique that has proven useful for malignant spinal tumors is stereotactic body radiation therapy; however, there is no literature to date supporting its use in GCTS.

With regard to radiation-induced sarcoma, these are often aggressive osteosarcomas. In the previously mentioned study by Leggon et al. they found 11% of their patients with sacral or pelvic GCT who received RT developed radiation-induced sarcomas at a mean of 9.1 years after RT [7]. Other studies have highlighted the risk of malignant transformation in patients with GCT who underwent high doses of RT (exceeding 40 Gy in total) [6,23]. RT should therefore only be considered for patients who are not surgical candidates or for whom no feasible surgical or alternative adjuvant options are available.

#### 4.5.2. Surgical Treatment of GCTS of the Lumbar Spine

Surgery remains the gold standard for treatment of GCTS despite recent publications on the role of denosumab as a stand-alone treatment. The goal of surgery is to remove as much tumor burden as possible, decompress neural elements at risk, and provide a stable spine. Published literature supports the total en bloc spondylectomy as the most definitive treatment with the lowest risk of recurrence when compared with intralesional curettage. The procedure can be performed through a posterior only or combined anterior-posterior approach as each individual case dictates based on WBB classification [9]. Stabilization usually consists of segmental stabilization above and below the operative level. The vertebral body is replaced with expandable or uniblock cages and can be supplemented by an anterior plate. The results and complications for different surgical techniques reported in literature are available for review in Supplemental Table S1 [6,7,8,9,10,22,47,48,49,50,51,52].

As surgical technology and techniques have advanced, new strategies for the removal of GCTS have been published in recent years. The role of neoadjuvant denosumab has expanded greatly. A 6-month pre-operative course of denosumab has been shown to aid in resection by reducing tumor burden and developing clear cortical margins for the tumor [53]. This strategy has shown promise in the en bloc resection of GCTS in several case reports and case series [11,39,49,50,54,55]. Three-dimensional printing has also been used as an aid in the treatment of GCTS. Lador et al. published the case of an L5 GCTS in an 18-year-old male which utilized a 3D model of the tumor for the selection of surgical approach and technique. Furthermore, a 3D printed implant was used to restore anatomic height and sagittal balance of the native spine [54].

**Figure 8:**
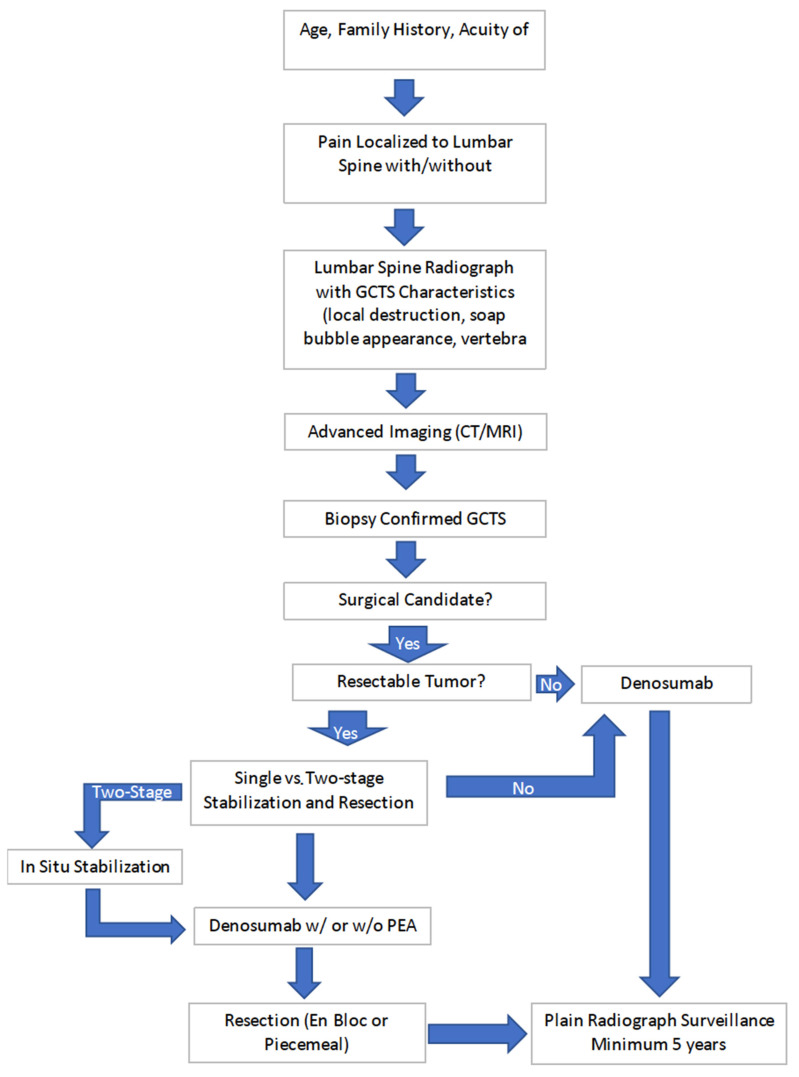
Proposed algorithm for treatment and diagnosis of GCT of the lumbar spine.

More recent innovations in the surgical treatment of GCTS include staged procedures. In this technique, the affected vertebral level is stabilized with percutaneous or open segmental instrumentation of the levels above and below the tumor. This stabilization provides pain relief and prevents progression of intralesional fractures for the 6-month duration of denosumab treatment. Prior segmental stabilization also helps to facilitate en bloc removal by removing the requirements for segmental stabilization during the staged resection procedure [50]. Our proposed diagnosis and treatment algorithm is presented below in Figure 8.

#### 4.5.3. Secondary Aneurysmal Bone Cysts

Aneurysmal bone cysts are benign expansile cystic lesions with an incidence of 1.4 cases in 100,000 people, accounting for 1% of all bone tumors and 15% of primary spine tumors [55,56]. An estimated 30–50% of all ABCs are superimposed on an already existing lesion, and are therefore described as secondary ABCs arising from the spine [57]. The most common primary lesion associated with secondary ABCs is GCTS [57]. Fluid-fluid levels seen on a CT and/or MRI are indicative of the formation of a secondary ABC. The fluid-fluid levels will be hypointense on T1 weighted imaging and hyperintense on T2 with contrast enhancement of the septa [57]. Secondary ABCs are typically aggressive and have a higher rate of recurrence and this should be taken into consideration while planning a treatment strategy. An 11-patient case series performed by Wu et al. showed a higher rate of recurrence with intralesional (3/4) vs. total spondylectomy (1/11) which indicates a more aggressive treatment strategy is required for the treatment of GCTS complicated by secondary ABC [57]. Long-term data on the efficacy of denosumab in the treatment of ABC are limited; however, a comprehensive review published by Alhumeid of 12 studies including 30 patients showed a radiographic response in 28/30 patients. However, recurrence was reported in 5/24 patients who completed or stopped the denosumab treatment [58]. In conclusion, given the high rate of recurrence after intralesional curettage and early promising data regarding the use of denosumab, we would recommend total en bloc spondylectomy with or without adjuvant denosumab in the treatment of GCTS complicated by secondary ABC.

#### 4.5.4. Diagnosis and Management of Recurrent Giant Cell Tumor

Despite available adjuvant and surgical treatments for GCTS, recurrence of GCTS is a known phenomenon which has proven difficult to treat. The incidence of recurrence ranges from 22 to 42% [7]. However, the true recurrence rate is difficult to determine due to inconsistencies in treatment method, both surgical and adjuvant, across several small case series.

Boriani et al. published a 49-patient case series with the goal of establishing risk factors for recurrence. They found that recurrence occurred in 11/49 cases (22%). Recurrence was associated with age < 25 years, surgical treatment performed, and Enneking stage of the tumor. No Enneking Stage II tumors recurred regardless of the operation performed. For Enneking Stage III tumors, 62% of tumors which underwent intralesional resection recurred vs. 9% in the en bloc spondylectomy group. Of interest, the 1 and 5 year recurrence free survival rate for Enneking Stage III treated with intralesional resection was 54% and 39%, respectively, while those treated with en bloc resection had a 100% and 90% recurrence free survival rate at 1 and 5 years, respectively [5].

Xu et al. published a case series of 102 patients diagnosed with GCT of the mobile spine. They calculated an overall recurrence rate of 37% (38/102). The study was broken into three treatment groups: total en bloc spondylectomy, piecemeal spondylectomy, and subtotal spondylectomy. The total en bloc spondylectomy group had a recurrence rate of 9% (1/11). The piecemeal spondylectomy group had a recurrence rate of 28% (7/25). The subtotal spondylectomy group performed considerably worse with a 45% (30/66) recurrence rate [7]. This evidence further supports that en bloc spondylectomy provides a better disease-free outcome, with total en bloc spondylectomy as the ideal surgical technique whenever feasible. The overall recurrence rate increased from 23.5% at 2 years follow up to 37% at 5 years follow up [7]. These data indicate that surveillance for reoccurrence should span at least 5 years after the index procedure.

## 5. Conclusions

The diagnosis of GCTS of the lumbar spine should proceed as follows. A clinical history of lower back pain with or without neurologic symptoms can be attributed to a wide range of pathology. After obtaining a comprehensive history and physical exam, the diagnosis should begin with radiographic imaging of the spine. Evidence of a locally aggressive and lytic lesion within the spine gives rise to a differential diagnosis including but not limited to GCTS and ABC. Advanced imaging such as a CT scan and MRI can both aid in the final diagnosis as well as help determine appropriate treatment. The final diagnosis of GCTS should be confirmed with a needle-core biopsy performed at the same institution as the planned resection.

The development of denosumab as an adjuvant therapy in GCT of the bone has had a profound impact on the surgical resection of GCTS. The ability to reduce the tumor burden and induce consolidation through the formation of cortical bone has increased the ease and effectiveness of both intralesional and total en bloc spondylectomy of affected vertebrae. More research will be required to determine the effectiveness and safety of denosumab as a long-term stand-alone treatment for GCTS. There remains no consensus on the appropriate duration of denosumab therapy at this time.

Advances in surgical technology such as 3D printing provide an interesting tool to facilitate the removal and subsequent reconstruction of GCTS. To date, we know of only one recorded case where this technology was used. Future studies will be required to assess whether or not the added cost of 3D printed models and implants provides a clinically significant benefit. Research into both denosumab as a stand-alone therapy and the use of 3D printing technology in the field of GCTS is difficult due to the relatively low incidence of the condition. Randomized controlled trials are difficult to perform due to the low number of subjects and the necessity to involve multiple tertiary centers in such studies. We hypothesize that a meta-analysis of case reports and case series will likely provide the strongest level of evidence for future therapies.

## Supplementary Materials

The following supporting information can be downloaded at: https://www.mdpi.com/article/10.3390/diagnostics12040857/s1, Table S1: Summary of Surgical Treatment Studies.

## Appendix A

**Table A1:** Table of all articles cited in the text with level of evidence.

Type	Reference #	Author	Publication Year	Title	Publishing Journal	Article Type	Level of Evidence
Journal Article	[40]	T. L. Aghaloo; A. L. Felsenfeld; S. Tetradis	2010	Osteonecrosis of the Jaw in a Patient on Denosumab	Journal of Oral and Maxillofacial Surgery	Case Report	4
Journal Article	[1]	P. Anract; G. De Pinieux; P. Cottias; P. Pouillart; M. Forest; B. Tomeno	1998	Malignant giant-cell tumours of bone	International Orthopaedics	Retrospective Case Series	3
Journal Article	[29]	J. Aoki; H. Tanikawa; K. Ishii; G. S. Seo; O. Karakida; S. Sone; T. Ichikawa; K. Kachi	1996	MR findings indicative of hemosiderin in giant-cell tumor of bone: frequency, cause, and diagnostic significance	AJR Am J Roentgenol	Retrospective Case Series	3
Journal Article	[5]	S. Boriani; S. Bandiera; R. Casadei; L. Boriani; R. Donthineni; A. Gasbarrini; E. Pignotti; R. Biagini; J. H. Schwab	2012	Giant cell tumor of the mobile spine: a review of 49 cases	Spine (Phila Pa 1976)	Retrospective Case Series	3
Journal Article	[11]	S. Boriani; R. Cecchinato; F. Cuzzocrea; S. Bandiera; M. Gambarotti; A. Gasbarrini	2020	Denosumab in the treatment of giant cell tumor of the spine. Preliminary report, review of the literature and protocol proposal	European Spine Journal	Retrospective Case Series	3
Journal Article	[4]	S. Boriani; J. N. Weinstein; R. Biagini	1997	Primary bone tumors of the spine. Terminology and surgical staging	Spine (Phila Pa 1976)	Literature Update	N/A
Journal Article	[23]	M. Campanacci; N. Baldini; S. Boriani; A. Sudanese	1987	Giant-cell tumor of bone	JBJS	Retrospective Case Series	3
Journal Article	[43]	A. Chakravarti; I. J. Spiro; E. B. Hug; H. J. Mankin; J. T. Efird; H. D. Suit	1999	Megavoltage radiation therapy for axial and inoperable giant-cell tumor of bone	J Bone Joint Surg Am	Retrospective Case Series	3
Journal Article	[47]	B. Z. Chin; T. Ji; X. Tang; R. Yang; W. Guo	2019	Three-Level Lumbar En Bloc Spondylectomy with Three-Dimensional−Printed Vertebrae Reconstruction for Recurrent Giant Cell Tumor	World Neurosurgery	Case Report	4
Journal Article	[48]	R. A. de Carvalho Cavalcante; R. A. Silva Marques; V. G. dos Santos; E. Sabino; J. A. C. Fraga; V. A. Zaccariotti; J. B. Arruda; Y. B. Fernandes	2016	Spondylectomy for Giant Cell Tumor After Denosumab Therapy	Spine (Philadelphia, Pa. 1976)	Case Report	4
Journal Article	[32]	M. Dominkus; P. Ruggieri; F. Bertoni; A. Briccoli; P. Picci; M. Rocca; M. Mercuri	2006	Histologically verified lung metastases in benign giant cell tumours--14 cases from a single institution	Int Orthop	Retrospective Case Series	3
Journal Article	[24]	R. Donthineni; L. Boriani; O. Ofluoglu; S. Bandiera	2009	Metastatic behaviour of giant cell tumour of the spine	International Orthopaedics	Retrospective Case Series	3
Journal Article	[38]	T. Goldschlager; N. Dea; M. Boyd; J. Reynolds; S. Patel; L. D. Rhines; E. Mendel; M. Pacheco; E. Ramos; T. A. Mattei; C. G. Fisher	2015	Giant cell tumors of the spine: has denosumab changed the treatment paradigm?	Journal of Neurosurgery: Spine SPI	Prospective Case Series	2
Journal Article	[33]	R. Gupta; V. Seethalakshmi; N. A. Jambhekar; S. Prabhudesai; N. Merchant; A. Puri; M. Agarwal	2008	Clinicopathologic profile of 470 giant cell tumors of bone from a cancer hospital in western India	Ann Diagn Pathol	Retrospective Case Series	3
Journal Article	[6]	R. A. Hart; S. Boriani; R. Biagini; B. Currier; J. N. Weinstein	1997	A system for surgical staging and management of spine tumors. A clinical outcome study of giant cell tumors of the spine	Spine (Phila Pa 1976)	Retrospective Case Series	3
Journal Article	[22]	Q. Jia; G. Chen; J. Cao; X. Yang; Z. Zhou; H. Wei; T. Liu; J. Xiao	2019	Clinical features and prognostic factors of pediatric spine giant cell tumors: report of 31 clinical cases in a single center	The spine journal	Retrospective Case Series	3
Journal Article	[41]	D. C. Khan; S. Malhotra; R. E. Stevens; A. D. Steinfeld	1999	Radiotherapy for the treatment of giant cell tumor of the spine: a report of six cases and review of the literature	Cancer Invest	Retrospective Case Series	3
Journal Article	[49]	H. Kinoshita; S. Orita; T. Yonemoto; T. Ishii; S. Iwata; H. Kamoda; T. Tsukanishi; K. Inage; K. Abe; M. Inoue; M. Norimoto; T. Umimura; K. Fujimoto; Y. Shiga; H. Kanamoto; T. Furuya; K. Takahashi; S. Ohtori	2019	Successful total en bloc spondylectomy of the L3 vertebra with a paravertebral giant cell tumor following preoperative treatment with denosumab: a case report	Journal of Medical Case Reports	Case Report	4
Journal Article	[2]	J. W. Kwon; H. W. Chung; E. Y. Cho; S. H. Hong; S. H. Choi; Y. C. Yoon; S. K. Yi	2007	MRI findings of giant cell tumors of the spine	AJR Am J Roentgenol	Retrospective Case Series	3
Journal Article	[54]	R. Lador; G. Regev; K. Salame; M. Khashan; Z. Lidar	2020	Use of 3-Dimensional Printing Technology in Complex Spine Surgeries	World Neurosurgery	Retrospective Case Series	3
Journal Article	[45]	R. E. Leggon; R. Zlotecki; J. Reith; M. T. Scarborough	2004	Giant cell tumor of the pelvis and sacrum: 17 cases and analysis of the literature	Clin Orthop Relat Res	Retrospective Case Series	3
Journal Article	[39]	T. A. Mattei; E. Ramos; A. A. Rehman; A. Shaw; S. R. Patel; E. Mendel	2014	Sustained long-term complete regression of a giant cell tumor of the spine after treatment with denosumab	The Spine Journal	Case Report	4
Journal Article	[18]	W. M. Mendenhall; R. A. Zlotecki; M. T. Scarborough; C. P. Gibbs; N. P. Mendenhall	2006	Giant cell tumor of bone	Am J Clin Oncol	Literature Review	N/A
Journal Article	[50]	K. Minato; T. Hirano; H. Kawashima; T. Yamagishi; K. Watanabe; M. Ohashi; A. Ogose; N. Endo	2021	Minimally Invasive Spinal Stabilization with Denosumab before Total Spondylectomy for a Collapsing Lower Lumbar Spinal Giant Cell Tumor	Acta medica Okayama	Case Report	4
Journal Article	[16]	M. D. Murphey; C. L. Andrews; D. J. Flemming; H. T. Temple; W. S. Smith; J. G. Smirniotopoulos	1996	From the archives of the AFIP. Primary tumors of the spine: radiologic pathologic correlation	Radiographics	Literature Review	N/A
Journal Article	[28]	M. D. Murphey; G. C. Nomikos; D. J. Flemming; F. H. Gannon; H. T. Temple; M. J. Kransdorf	2001	From the archives of AFIP. Imaging of giant cell tumor and giant cell reparative granuloma of bone: radiologic-pathologic correlation	Radiographics	Literature Review	N/A
Journal Article	[19]	S. Orguc; R. Arkun	2014	Primary tumors of the spine	Semin Musculoskelet Radiol	Literature Review	N/A
Journal Article	[53]	E. Palmerini; E. L. Staals; L. B. Jones; D. M. Donati; A. Longhi; R. L. Randall	2020	Role of (Neo)adjuvant Denosumab for Giant Cell Tumor of Bone	Current Treatment Options in Oncology	Case Report	4
Journal Article	[26]	S. S. Randhawa; A. K. N. Kwan; C. K. Chiu; C. Y. W. Chan; M. K. Kwan	2016	Neurological Recovery in Two Patients with Cauda Equina Syndrome Secondary to L5 Lumbar Spine Giant Cell Tumour after Treatment with Denosumab without Surgery	Asian Spine J	Retrospective Case Series	3
Journal Article	[15]	Yonezawa N, Murakami H, Demura S, et al,	2019	Morphologic Changes After Denosumab Therapy in Patients with Giant Cell Tumor of the Spine: Report of Four Cases and a Review of the Literature	World Neurosurgery	Retrospective Case Series	3
Journal Article	[36]	M. Rock	1990	Curettage of giant cell tumor of bone. Factors influencing local recurrences and metastasis	Chir Organi Mov	Retrospective Case Series	3
Journal Article	[34]	M. G. Rock; D. J. Pritchard; K. K. Unni	1984	Metastases from histologically benign giant-cell tumor of bone	J Bone Joint Surg Am	Retrospective Case Series	3
Journal Article	[46]	F. Roeder; C. Timke; F. Zwicker; C. Thieke; M. Bischof; J. Debus; P. E. Huber	2010	Intensity modulated radiotherapy (IMRT) in benign giant cell tumors--a single institution case series and a short review of the literature	Radiat Oncol	Retrospective Case Series	3
Journal Article	[44]	P. Ruggieri; A. F. Mavrogenis; G. Ussia; A. Angelini; P. J. Papagelopoulos; M. Mercuri	2010	Recurrence after and complications associated with adjuvant treatments for sacral giant cell tumor	Clin Orthop Relat Res	Retrospective Case Series	3
Journal Article	[20]	B. K. Sanjay; F. H. Sim; K. K. Unni; R. A. McLeod; R. A. Klassen	1993	Giant-cell tumours of the spine	J Bone Joint Surg Br	Retrospective Case Series	3
Journal Article	[51]	D. R. Santiago-Dieppa; L. S. Hwang; A. Bydon; Z. L. Gokaslan; E. F. McCarthy; T. F. Witham	2014	L4 and L5 spondylectomy for en bloc resection of giant cell tumor and review of the literature	Evidence-based spine-care journal	Case Report	4
Journal Article	[42]	R. R. Sharma; A. K. Mahapatra; S. J. Pawar; J. Sousa; E. J. Dev	2002	Craniospinal giant cell tumors: clinicoradiological analysis in a series of 11 cases	J Clin Neurosci	Retrospective Case Series	3
Journal Article	[27]	L. S. Shi; Y. Q. Li; W. J. Wu; Z. K. Zhang; F. Gao; M. Latif	2015	Imaging appearance of giant cell tumour of the spine above the sacrum	The British journal of radiology	Retrospective Case Series	3
Journal Article	[52]	Y. Shimada; M. Hongo; N. Miyakoshi; Y. Kasukawa; S. Ando; E. Itoi; E. Abe	2007	Giant cell tumor of fifth lumbar vertebrae: two case reports and review of the literature	Spine J	Case Report	4
Journal Article	[25]	M.-J. Si; C.-G. Wang; C.-S. Wang; L.-J. Du; X.-Y. Ding; W.-B. Zhang; Y. Lu; J.-Y. Zu	2014	Giant cell tumours of the mobile spine: characteristic imaging features and differential diagnosis	La radiologia medica	Retrospective Case Series	3
Journal Article	[35]	K. A. Siebenrock; K. K. Unni; M. G. Rock	1998	Giant-cell tumour of bone metastasising to the lungs. A long-term follow-up	J Bone Joint Surg Br	Retrospective Case Series	3
Journal Article	[12]	D. Thomas; R. Henshaw; K. Skubitz; S. Chawla; A. Staddon; J.-Y. Blay; M. Roudier; J. Smith; Z. Ye; W. Sohn; R. Dansey; S. Jun	2010	Denosumab in patients with giant-cell tumour of bone: an open-label, phase 2 study	The Lancet Oncology	Prospective Cohort	2
Journal Article	[17]	A. J. Verschoor; J. Bovée; M. J. L. Mastboom; P. D. Sander Dijkstra; M. A. J. Van De Sande; H. Gelderblom	2018	Incidence and demographics of giant cell tumor of bone in The Netherlands: First nationwide Pathology Registry Study	Acta Orthop	Database Study	4
Journal Article	[21]	S. Wilartratsami; S. Muangsomboon; S. Benjarassameroj; R. Phimolsarnti; C. Chavasiri; P. Luksanapruksa	2014	Prevalence of primary spinal tumors: 15-year data from Siriraj Hospital	J Med Assoc Thai	Retrospective Case Series	3
Journal Article	[7]	W. Xu; X. Li; W. Huang; Y. Wang; S. Han; S. Chen; L. Xu; X. Yang; T. Liu; J. Xiao	2013	Factors Affecting Prognosis of Patients with Giant Cell Tumors of the Mobile Spine: Retrospective Analysis of 102 Patients in a Single Center	Annals of Surgical Oncology	Retrospective Case Series	3
Journal Article	[14]	T. Yayama; K. Mori; A. Nakamura; T. Mimura; S. Imai	2020	Denosumab Therapy for Giant-cell Tumor of the Lumbar Spine: A Case Report and Immunohistochemical Examination	Journal of orthopaedic case reports	Case Report	4
Journal Article	[31]	H. Yin; M. Cheng; B. Li; B. Li; P. Wang; T. Meng; J. Wang; W. Zhou; W. Yan; J. Xiao	2015	Treatment and outcome of malignant giant cell tumor in the spine	Journal of Neuro-Oncology	Retrospective Case Series	3
Journal Article	[8]	N. Yokogawa; H. Murakami; S. Demura; S. Kato; K. Yoshioka; T. Shimizu; N. Oku; R. Kitagawa; H. Tsuchiya	2018	Total spondylectomy for Enneking stage III giant cell tumor of the mobile spine	European Spine Journal	Retrospective Case Series	3
Journal Article	[15]	N. Yonezawa; H. Murakami; S. Kato; A. Takeuchi; H. Tsuchiya	2017	Giant cell tumor of the thoracic spine completely removed by total spondylectomy after neoadjuvant denosumab therapy	Eur Spine J	Case Report	4
Journal Article	[9]	H. Zhou; L. Jiang; F. Wei; M. Yu; F. l. Wu; X. g. Liu; Z. j. Liu	2018	Surgical approach selection for total spondylectomy for the treatment of giant cell tumors in the lumbar spine: A retrospective analysis of 12 patients from a single center	Asia-Pacific journal of clinical oncology	Retrospective Case Series	3
Journal Article	[10]	M. Zhou; H. Yang; K. Chen; G. Wang; J. Lu; Y. Ji; C. Wu; C. Chen; H. Hu	2013	Surgical treatment of giant cell tumors of the sacrum and spine combined with pre-operative transarterial embolization	Oncol Lett	Retrospective Case Series	3
